# Particulate matter emissions from biochar-amended soils as a potential tradeoff to the negative emission potential

**DOI:** 10.1038/srep35984

**Published:** 2016-10-26

**Authors:** Sujith Ravi, Brenton S. Sharratt, Junran Li, Stuart Olshevski, Zhongju Meng, Jianguo Zhang

**Affiliations:** 1 Department of Earth & Environmental Science, Temple University, Philadelphia, PA 19122, USA; 2 Northwest Sustainable Agroecosystems Research, USDA-ARS, Pullman, WA 99164, USA; 3 Department of Geosciences, The University of Tulsa, Tulsa, OK 74104, USA; 4 College of Desert Science and Engineering, Inner Mongolia Agricultural University, Inner Mongolia 010019, China; 5 College of Natural Resources and Environment, Northwest A&F University, Shaanxi 712100, China

## Abstract

Novel carbon sequestration strategies such as large-scale land application of biochar may provide sustainable pathways to increase the terrestrial storage of carbon. Biochar has a long residence time in the soil and hence comprehensive studies are urgently needed to quantify the environmental impacts of large-scale biochar application. In particular, black carbon emissions from soils amended with biochar may counteract the negative emission potential due to the impacts on air quality, climate, and biogeochemical cycles. We investigated, using wind tunnel experiments, the particulate matter emission potential of a sand and two agriculturally important soils amended with different concentrations of biochar, in comparison to control soils. Our results indicate that biochar application considerably increases particulate emissions possibly by two mechanisms–the accelerated emission of fine biochar particles and the generation and emission of fine biochar particles resulting from abrasion of large biochar particles by sand grains. Our study highlights the importance of considering the background soil properties (e.g., texture) and geomorphological processes (e.g., aeolian transport) for biochar-based carbon sequestration programs.

A multitude of integrated assessment models of future climate mitigation pathways stress the need for large-scale negative emission technologies that result in the net removal of carbon dioxide from the atmosphere^1^. Agriculture (croplands and pastures) accounts for the largest share of earth’s terrestrial surface (~40%) and is recognized as a major contributor to anthropogenic greenhouse gas emissions^2^,^3^. In this regard, novel carbon sequestration strategies such as large-scale application of biochar to agricultural soils may provide sustainable pathways to increase the terrestrial storage of carbon with simultaneous co-benefits including sustainable bioenergy production and improvement of soil and water quality^4^,^5^,^6^. Recently, large-scale application of biochar has been promoted as a strategy for reclaiming degraded landscapes including areas affected by accelerated soil erosion and salinization^7^.

Biochar is a highly stable carbon-rich porous substance produced by the pyrolysis of organic material including agricultural residues, feedstock residues from biofuel production, and manures^8^,^9^. The properties and benefits of biochar depend on the production process and the intended use^9^. Large-scale application of biochar in managed ecosystems to sequester atmospheric carbon is considered to be a sustainable “negative emission technology”^9^,^10^,^11^. In agricultural soils, the application of biochar in appropriate quantities is known to increase crop productivity potentially by improving water and nutrient retention properties^12^,^13^,^14^. Furthermore, biochar application is widely used for bioremediation in soils affected by contamination^15^,^16^,^17^. Biochar particles actively remove heavy metal ions, organic compounds and microbes from soil, through a variety of physio-chemical processes including electrostatic interactions, sorption, partition, and formation of complexes^18^.

Due to recalcitrant nature of the organic carbon in biochar, the applied biochar is expected to last hundreds of years in soil^18^,^19^. Residence time calculation in the previous studies do not account for carbon loss due to soil erosion by wind and water, partly because the erosion potential of biochar and downstream impacts are unknown. This is a critical research gap, as climate change (e.g., increase in aridity, recurrent droughts), disturbances (e.g., fires, grazing), and lack of proper soil conservation measures have rendered soil in many agricultural and rangeland systems highly susceptible to accelerated soil erosion by wind^20^,^21^,^22^. As fine biochar particles effectively adsorb/trap contaminants and pathogens from the soil, the preferential erosion of fine biochar particles by wind may lead to concentration of contaminants in the airborne dust. The contaminants on biochar particles are mostly bioavailable, meaning that the contaminants can be desorbed and released to biological systems. Hence the biochar-loaded dust from contaminated agricultural areas may be a potential health hazard. Once in the atmosphere, black carbon particles absorb short wave solar radiation and alter the properties and distribution of clouds, thereby impacting hydrological cycle and climate^23^,^24^. Thus, the particulate matter emissions (e.g., black carbon) from biochar-amended soils may counteract the negative emission potential^24^.

Considering the potential impacts of black carbon aerosols on air quality, climate, radiation budgets and biogeochemical (carbon) cycles, comprehensive studies are urgently needed to assess the emission potential of biochar-amended soils^10^,^24^. Even though several studies have investigated the impacts of biochar application on a variety of soil properties, very few studies have investigated the impacts on particulate matter emissions. In particular, we are not aware of any studies that have examined wind (aeolian) erosion and subsequent particulate emissions from biochar-amended soils. Here, using laboratory wind tunnel experiments, we investigated the particulate matter emission potentials of biochar-amended soils. We mixed biochar (unsieved or sieved to appropriate particle sizes and application rates of 5, 10 and 20% of the soil by volume) to a pure sand (Ottawa sand) and two agriculturally important soils (Ritzville silt loam and Warden sandy loam) and estimated the changes in fine particulate matter emissions (PM_10_ or particulate matter ≤10 μm in diameter) in comparison to control soils.

## Results

### Particulate matter emissions

**Figure 1:**
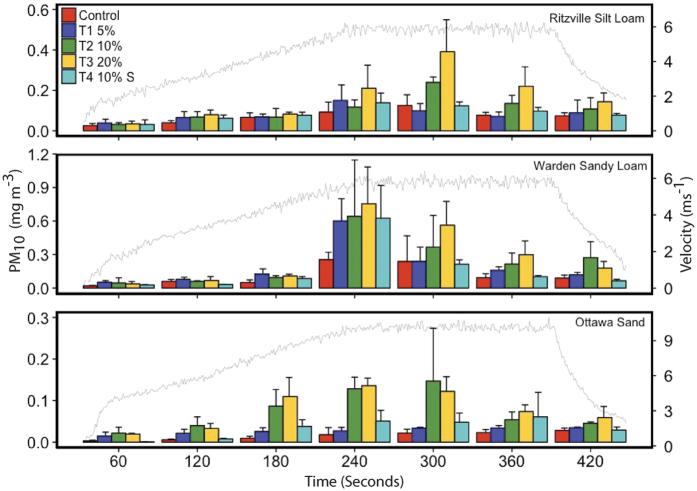
PM_10_ concentrations from the control and the treatments for the sand and two soil types during the seven-minute wind tunnel experiments. Average values and standard deviations were calculated after every minute. Typical wind velocities (measured at 100 mm height) for the sand and two soil types during the tests are shown in gray lines.

The PM_10_ concentration (at 10 mm from the soil surface) was averaged over each subsequent minute to show the changes in particulate matter emission during each wind tunnel test (Fig. 1). The average PM_10_ emissions were generally higher from biochar-amended soils (T1, T2, T3) versus non-amended soils (Control) even during the initial phase of the wind tunnel test (pre-threshold velocity conditions). There was a large increase in PM_10_ emissions when the velocity attained threshold conditions, especially for all the biochar-amended soils (Fig. 1). The PM_10_ emissions were lower for the soils amended with sieved biochar (T4) compared to the soils amended with unsieved biochar (T2). However, the emissions from soils amended with sieved biochar (T4) were lower than control soils (Control) in the initial stages and higher when threshold conditions were attained (Fig. 1). All controls and treatments showed a decline in PM_10_ emission at later stages of the wind tunnel tests even when the wind velocity was higher than the threshold for emissions, possibly due to limited supply of fine particles and abraders.

### Average particulate matter flux

**Figure 2:**
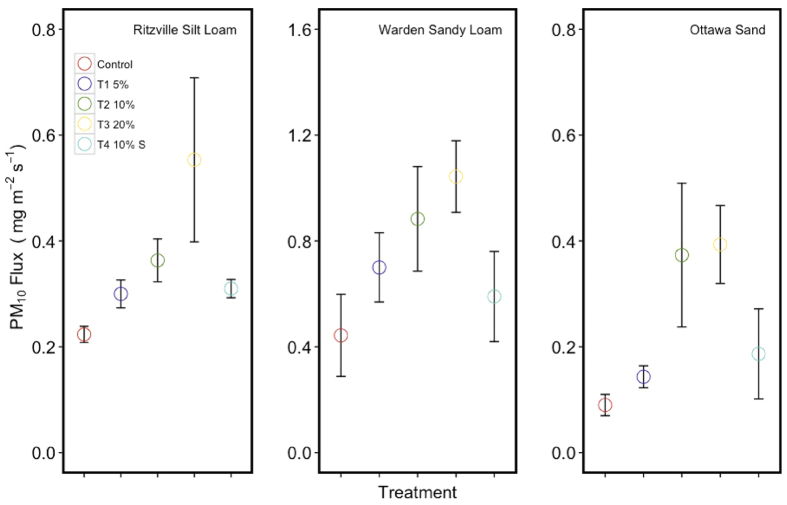
Average PM_10_ flux of particulate matter (at 10 mm height) generated by the control and the treatments for the sand and two soil types during the wind tunnel tests.

Horizontal particulate matter flux from different treatments was determined from PM_10_ concentration and wind velocity at the 10 mm height. The average PM_10_ flux for the seven-minute test indicated an increase in average PM_10_ flux with increasing biochar concentration for all the soils used in the experiment (Fig. 2). Statistical tests (ANOVA) indicated significant differences between control and treatments (p < 0.001) and between soil types (p < 0.001). Tukey HSD posthoc tests showed significant differences between the control and all the treatments (p < 0.001) except the T4 (10% Sieved). See supplementary for more information.

### Threshold shear velocity and soil moisture

**Figure 3:**
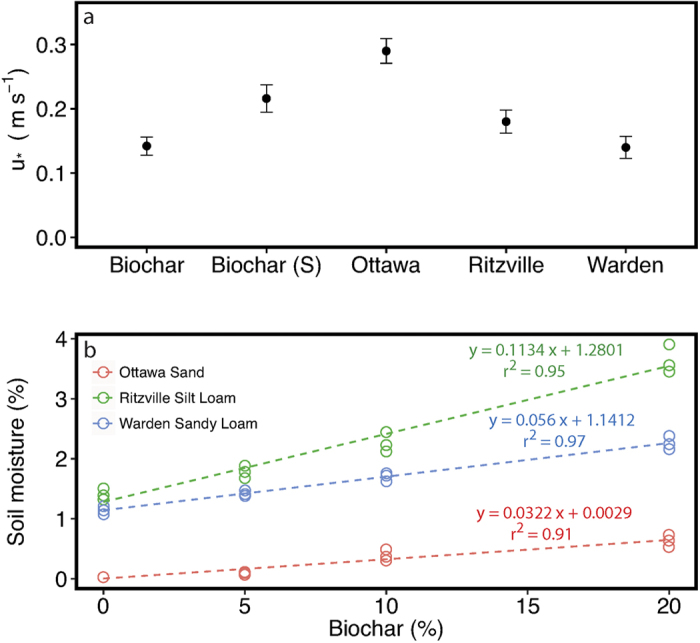
(a) Threshold shear velocity of the control sand, soils and biochar used for the study, (b) Changes in gravimetric soil moisture content as a result of different levels of biochar amendments for the sand and two soils used in this study.

The threshold shear velocity, the minimum velocity for wind erosion to occur, was highest for the control Ottawa sand and lowest for the control Warden sandy loam (Fig. 3a). The estimated threshold velocities for the two soil types are comparable to those estimated from previous wind tunnel experiments^25^. We were unable to determine threshold velocity of biochar-amended soils due to the difficulty in differentiating between the initial increase in PM_10_ emissions from fine biochar (or the resuspension of dust without saltation) and the background soil. Gravimetric soil moisture measurements indicate that the addition of biochar considerably increased moisture content of all the soils used for this study. Soil moisture content increased with an increase in biochar concentration and the rate of increase was higher for Ritzville silt loam compared to Warden sandy loam and Ottawa sand (Fig. 3b). The average moisture content of air-dried biochar was around 30%.

## Discussion

The increase in water–holding capacity with the addition of high surface area biochar particles to soils was expected to decrease wind erosion and subsequent PM_10_ emissions by increasing the interparticle forces (due to moisture bonding) in soils^21^,^26^. Interestingly, our results indicate that biochar addition significantly increased the average PM_10_ concentrations from all the soil types used in this study. For pre-threshold velocity conditions, the increase in PM_10_ emissions from biochar-amended soils can be attributed to the presence of very fine biochar particles, which are lighter and less dense than the bulk soil and can be easily entrained by wind. This resuspension of fine particles occurring without saltation is thought to be a significant mechanism of contaminant transport into the atmosphere^27^. After threshold velocity conditions are attained, the emissions were further generated by the abrasion of low-density large biochar particles by sand grains. This process, referred to as “saltation bombardment”, maintained the higher particulate matter emissions from biochar-amended soils even after the fine biochar particles were depleted. The effect is clear in the case of higher PM_10_ emissions from soils amended with sieved (>2mm) biochar in the post-threshold phase (Fig. 1). In biochar amended agricultural systems, a fraction of fine black carbon particles generated by abrasion may be deposited back into the soil and may be easily transported again by wind or moved to groundwater by infiltration and leaching^28^.

Our study highlights the importance of considering the threshold shear velocity and physical properties (the availability of saltation-sized sand grains) of soil before amending with biochar to minimize any impact on air quality. The Warden sandy loam was characterized by the presence of more saltation-sized sand particles and had a lower threshold velocity than other soil types and the biochar, which explains the higher PM_10_ emissions. Even though Ottawa sand had sand particles capable of abrasion, the particles were larger and hence the threshold velocity was higher. The particle size, particle density and interparticle forces (*F*_*i*_) due to moisture affect the threshold shear velocity (u_*_) of saltation as:


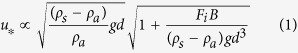


where *ρ*_*s*_ and *ρ*_*a*_ are densities of soil and air, *d* is the diameter of particle, *g* is the acceleration due to gravity, *F*_*i*_ represents interparticle forces, and *B* is a constant^29^,^30^. The first part of the expression for u_*_ accounts for particle diameter and density and the second part accounts for the interparticle forces due to moisture (adsorption and capillary forces) between soils, which become increasingly important in finer soils^31^. In our experiments using air dried soils (capillary forces are negligible), an expected increase in interparticle forces resulting from increased water adsorption due to biochar addition is counteracted by an increase in finer (lower diameter) and less denser (approximately twice lower) biochar particles. This explains the decrease in threshold shear velocity and increase in PM_10_ emissions with increases in biochar concentration.

**Figure 4:**
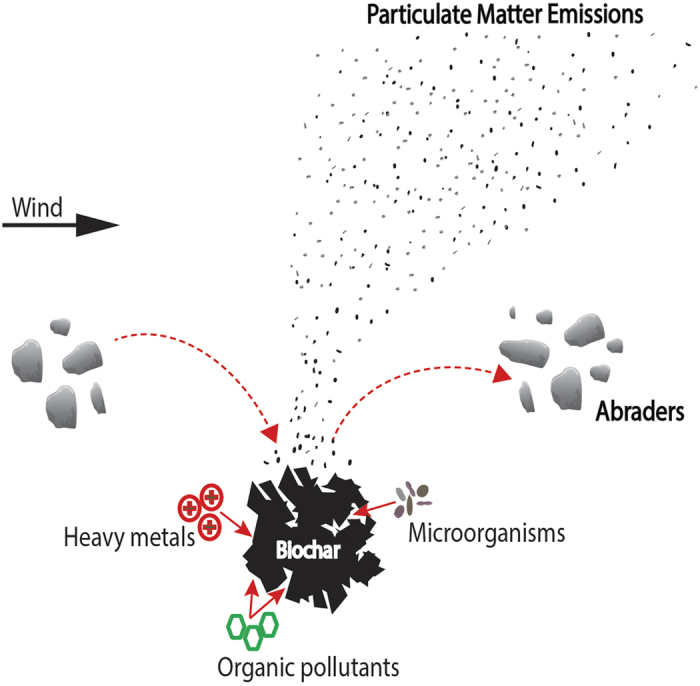
A conceptual model of particulate matter emissions from biochar-amended soils.

Biochar particles effectively adsorb/trap contaminants and pathogens from the soil and the emissions of particulate matter (fine biochar particles) may lead to concentration of contaminants in the airborne dust (Fig. 4). Several studies have recommended the use of large biochar pellets to limit black carbon emissions from amended soils by decreasing the potential emission of very fine particles^29^,^32^. However, our study experimentally demonstrated that abrasion of large biochar particles by sand particles may result in sustained emissions from biochar amended soils, even after the initial removal of very fine black carbon particles (Figs 1 and 4). Biochar application is generally recommended for soil quality improvement programs in less fertile sandy soils, which provides a consistent supply of abraders to increase black carbon emissions.

The biochar particles are highly sensitive to changes in atmospheric humidity due to their high surface area^18^ and hence the effect of air humidity on the susceptibility of biochar-amended soils needs to be considered^31^. The particulate matter emissions are expected to increase substantially under drier scenarios, which are predicted to be more frequent in many areas in the world in the future^26^,^33^. For our study, we used a commercially available biochar product and thus acknowledge that the results may vary depending on the biochar properties. Furthermore, the samples used in our study may not replicate the complex biochar-soil interactions under field conditions, which evolve with time. Nevertheless, our experiment represents a first step towards understanding the emission potential of soils following biochar application in short time-scales (days of weeks after biochar application).

Future biochar application scenarios estimate that up to 101.5 Pg C from biochar will be applied to 4.03 G ha of cropland and pastures globally in the next 100 years^32^. The applied biochar has a long residence time in the soil, and hence there is an urgent need to understand the stability and transformation of biochar in the soil to investigate the long-term environmental impacts. The majority of existing studies have focused on bio-chemical and surface processes involved in the disintegration of applied biochar particles, while our experimental studies highlight the importance of physical and geomorphological processes in natural systems. Our results demonstrate for the first time, that biochar addition significantly increases particulate matter emissions from the two soils and the sand studied, either by emission of fine particles, or by generation and emission of fines by abrasion of large biochar particles (Fig. 4). Thus the physical degradation by abrasion is a significant but poorly understood process of generation and removal of fine black carbon particles from biochar-amended soils. Considering the impact of black carbon aerosols for air quality and global climate, the emissions resulting from biochar-amended soils and their downwind impacts are important factors to consider in biochar-based carbon sequestration, remediation and soil quality improvement programs.

## Methods

### General characterization of soils

A pure silica sand and two soils were used for the study: Ottawa sand, Warden sandy loam, and Ritzville silt loam. Ottawa sand is a pure unground silica sand (US Silica, IL, USA) and is commonly used in many geophysical experiments. Ritzville silt loam and Warden sandy loam occur in the low precipitation zones (200–300 mm year^−1^) of the Columbia Plateau (WA, USA). These soils are agriculturally important and are highly susceptible to wind erosion. The commercially available biochar product used in this study was manufactured by slow pyrolysis of woody feedstock with an organic carbon content of ~86% and a bulk density of 0.20 g cm^−3^. The biochar produced from woody feedstock (pine) by slow pyrolysis at low temperatures (300**°**C) typically has a particle density of around 1.3 g cm^−3 ^34. The particle size distribution of the sand and two soils were determined using a laser diffraction particle size analyzer (Beckman Coulter, LS 13 320*). This analyzer measures the grain size distribution of particles suspended in a liquid using the principles of light scattering and has a dynamic measurement range of 0.017 to 2000 *μm*. The particle size distribution of the biochar was determined using a CAMSIZER (Retsch Technology Gmbh, Haann, Germany*) dry particle size analyzer, which comprehensively characterizes dry free flowing bulk materials by digitally imaging and analyzing thousands of particles of each sample with a measurement range of ~0.04 mm to 8 mm.

The air-dried soil samples were gently crushed to remove large aggregates and clods, and then sieved through a 2 mm sieve a few days before the wind tunnel experiments. Three concentrations of the air-dried biochar were applied to the sand and two soils to represent typical biochar application rates in soils. The target rates were 5, 10, and 20% biochar by volume. In addition to the three application treatments, coarse biochar (>2 mm in diameter) was also applied to each soil type at a concentration of 10% by volume. More information of the soils and biochar used in this study may be found in S1 of the Supplementary Information.

### Wind tunnel experiments

The non-recirculating wind tunnel used for this study (USDA-ARS Colombia Plateau Air Quality Research, Pullman, WA, USA) is 7.3 m long, 1.0 m wide, and 1.2 m high^25^. The test section is equipped with removable metal trays (0.2 m wide × 0.02 m deep × 1 m long) that mount flush with the floor of the tunnel. The wind tunnel engine has 13 throttle settings, which can generate free stream wind velocities of 2 to 20 m s^−1^. The floor of the tunnel is constructed of plywood coated with sand paper with similar roughness as the soil surface in the tray. Climatic parameters (e.g., atmospheric humidity and temperature) were not regulated for these experiments. The experiments were carried out over a temperature range of 10–20 °C and relative humidity of 40–50%.

The wind velocity was measured using differential pressure transducers connected to pitot tubes installed downwind from the soil tray at six different heights (5, 10, 20, 30, 50, and 100 mm) from the surface of the tray. Atmospheric pressure, air temperature and relative humidity were monitored at the entrance of the working section and used along with differential pressure measurements to calculate wind velocity. Fine particulate matter (PM_10_ or particulate matter ≤10 μm in diameter) concentrations during the wind tunnel experiments were measured at the downwind edge of the soil tray using a DustTrak aerosol monitor (Model 8520, TSI incorporated, Shoreview, MN, USA*) mounted at 10 mm height. Two additional DustTrak monitors were used to monitor background (ambient) PM_10_ concentration. The data were collected every second (1 Hz frequency) during the wind tunnel tests using a datalogger. The design and operation of the wind tunnel were discussed in *Sharatt and Vaddella* (2012)^25^.

The experimental design included three replicates for each treatment. The soil and biochar were exposed to ambient conditions in the wind tunnel facility for 24 hours before each test. Appropriate quantities of biochar and soil were thoroughly mixed together in small batches and filled in the test trays. The soil and biochar-filled trays were allowed to equilibrate with the ambient atmospheric humidity (~30 minutes) before placing the tray into the wind tunnel. The gravimetric moisture content in the upper 2 mm of the soil profile was measured after each test, while simultaneous measurements of gravimetric soil moisture were made in a control tray for each soil type. The wind velocity was carefully controlled to provide the comparable wind velocity profiles for the treatments and replicates of each soil. The wind speed was initially increased stepwise by adjusting the engine throttle setting until a threshold velocity was attained. Wind speed was then increased to the next throttle setting (above the threshold velocity) for an additional three minutes. The duration of each wind tunnel test was 7 minutes. DustTrak aerosol monitors were used to determine the threshold velocity, which was determined as the average velocity of 5 seconds before and after the point at which an abrupt increase in PM_10_ concentration was observed. Shear velocity (u_*_) was determined from the wind speed profile according to the Prandtl-von Karman law^26^. Statistical tests were conducted (one-way ANOVA, R ver. 3.2.4, 2016) to identify differences in PM_10_ flux among treatments. See Supplementary Information S2 for more information on the statistical analysis.

## Additional Information

**How to cite this article**: Ravi, S. *et al*. Particulate matter emissions from biochar-amended soils as a potential tradeoff to the negative emission potential. *Sci. Rep.* **6**, 35984; doi: 10.1038/srep35984 (2016).

**Publisher’s note:** Springer Nature remains neutral with regard to jurisdictional claims in published maps and institutional affiliations.

## Supplementary Material

Supplementary Information
