# Supplementary material for: Particulate matter emissions from biochar-amended soils as a potential tradeoff to the negative emission potential
Source: Sci Rep. 2016 Oct 26;6:35984. doi: 10.1038/srep35984 (PMC5080604; doi:10.1038/srep35984)
Supplement: Supplementary Information [file srep35984-s1.doc]

**Particulate matter emissions from biochar-amended soils as a potential tradeoff to the negative emission potential**

**Supplementary information**

Sujith Ravi^a^*^†^*, Brenton S. Sharratt*^b^*, Junran Li*^c^*, Stuart Olshevski*^a^*, Zhongju Meng*^d^* and Jianguo Zhang*^e^*

*^a^* Department of Earth & Environmental Sciences, Temple University, Philadelphia, PA 19122, *USA. Tel: 00 1703 581 8186; E-mail:* [*sravi@temple.edu*](mailto:sujith@stanford.edu)

*^b^* Northwest Sustainable Agroecosystems Research, USDA-ARS, Pullman, WA 99164.

*^c^* Department of Geosciences, The University of Tulsa, Tulsa, OK 74104.

*^d,b^* College of Ecology and Environmental Science, Inner Mongolia Agricultural University, Inner Mongolia, China.

*^e,b^* College of Natural Resources and Environment, Northwest A&F University, Shaanxi, China.

*^†^ Corresponding author*

**S1: Supplementary information**

**Soil and Biochar characteristics**

A pure sand and two soils were used for the study: Ottawa sand, Warden sandy loam and Ritzville silt loam. Ottawa sand is a pure unground silica sand (Ottawa sand, ASTM 20/30, US Silica, IL, USA) and is commonly used in many geophysical experiments because of its uniform grain size (97% of grains are 0.60 - 0.85 mm in diameter) and spherical particles. Ritzville silt loam and Warden sandy loam occur in the low precipitation zones (200 -300 mm year^-1^) of the Colombia Plateau (WA, USA). These agriculturally important soils respectively comprise 9.7 and 3.1% of the land in the Columbia Plateau and are highly susceptible to wind erosion. Ritzville silt loam is coarse textured, mixed superactive, mesic calcidic Haploxerolls and was collected near Ritzville, WA (47**°** 08’N, 118**°** 28’W). Warden sandy loam is coarse-silty, mixed superactive mesic seric Haplocambids and was collected near Paterson, WA (46**°** 01 N, 119**°** 37’W). These soils were historically managed in a winter wheat – summer fallow rotation.

The commercial biochar product used in this study was produced by slow pyrolysis of woody feedstock with an organic carbon content of ~86% (Confluence Energy, Kremmling, CO, USA*). The biochar produced from woody feedstock (pine) by slow pyrolysis at low temperatures (300**°**C) typically has a particle density of 1.3 gcm^-3^. The particle size distribution of the sand (Figure S1.1) and two soils (Figure S1.2) were determined using a LS 13320 laser diffraction-particle size analyzer (Beckman Coulter, Inc. CA, USA*). The particle size distribution of the biochar was determined using a CAMSIZER (Retsch Technology Gmbh, Haann, Germany*) dry particle size analyzer, which comprehensively characterizes dry free flowing bulk materials by digitally imaging and analyzing thousands of particles of each sample (Figure S1.3).

**Table1:** Characteristics of test materials

| **Test materials** | **Source** |  |
| --- | --- | --- |
|  |  | **Median Diameter (D_50_)**  *μm* |
| Ottawa sand | US Silica, IL | 908 |
| Warden sandy loam | Field  46° 01 N, 119° 37’W  Paterson, WA | 93 |
| Ritzville silt loam | Field  47° 08’N, 118° 28’W  Ritzville, WA | 47 |
| Biochar -  Unsieved | Confluence Energy, CO | 2603 |
| Biochar -Sieved | Confluence Energy, CO | 3688 |

***
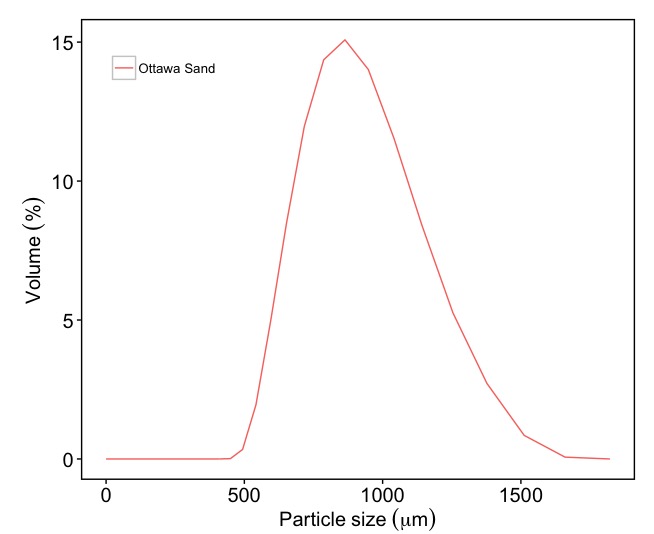
***

**Figure S1.1:** The particle size distribution of Ottawa sand determined using a laser diffraction particle size analyzer.

**
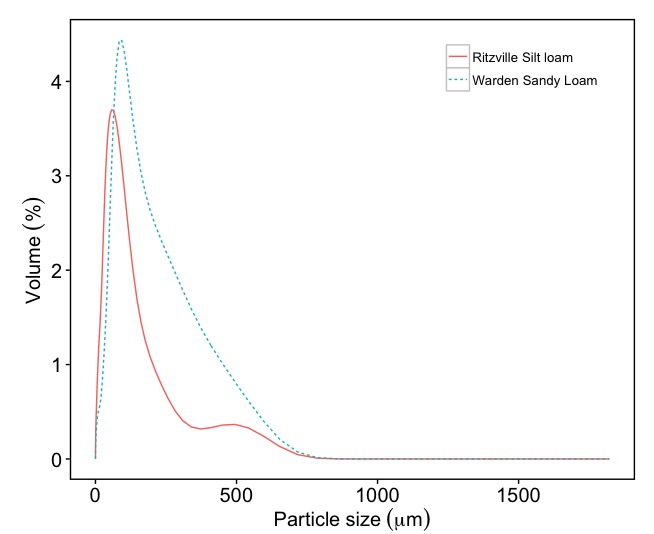
**

**Figure S1.2:** The particle size distribution of Ritzville Silt Loam and Warden Sandy Loam determined using a laser-diffraction particle size analyzer.

***
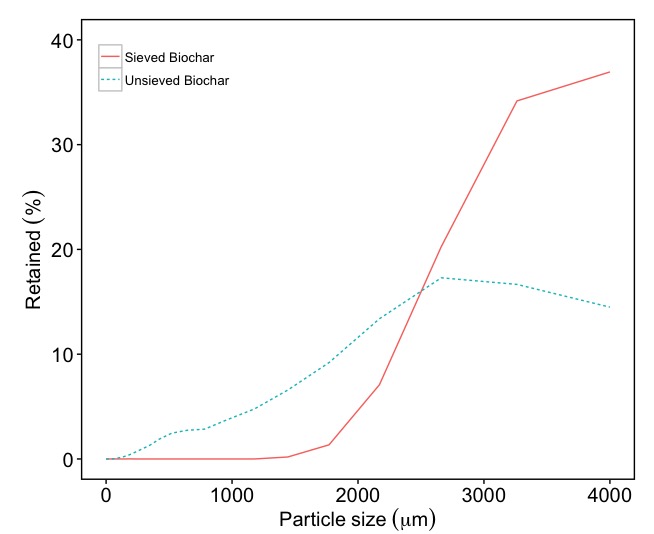
***

**Figure S1.3:** The particle size distribution of sieved and unsieved biochar determined using a CAMSIZER (Retsch Technology, Gmbh)

**S2: Supplementary information**

**Statistical analysis**

Statistical tests were conducted (one-way ANOVA, R ver. 3.2.4, 2016) to identify differences in PM_10_ flux among soil types and treatments. Statistical tests (ANOVA) indicated significant differences between control and treatments (p< 0.001) and between soil types (p<0.001). Tukey HSD posthoc tests showed significant differences between the control and all the treatments (p<0.001) except the T4 (10% Sieved). Normality of residuals was ensured using log transformation.

Tukey multiple comparisons of means

95% family-wise confidence level

Fit: aov(formula = log(PM10) ~ Soil * Treatment, data)

**Soil Types**

**diff lwr upr p adj**

Ritzville-Ottawa 0.5172758 0.2939551 0.7405965 9.2e-06

Warden-Ottawa 1.2464398 1.0231191 1.4697605 0.0e+00

Warden-Ritzville 0.7291640 0.5058433 0.9524847 0.0e+00

**Treatments**

**diff lwr upr p adj**

T1 - C 0.4222938 0.08307676 0.76151089 0.0089909

T2 - C 0.8659128 0.52669574 1.20512987 0.0000003

T3 - C 1.0886007 0.74938366 1.42781779 0.0000000

T4 - C 0.4349318 0.09571476 0.77414889 0.0068120

T2 - T1 0.4436190 0.10440191 0.78283604 0.0056178

T3 - T1 0.6663069 0.32708983 1.00552396 0.0000304

T4 - T1 0.0126380 -0.32657907 0.35185506 0.9999672

T3 - T2 0.2226879 -0.11652914 0.56190499 0.3370340

T4 - T2 -0.4309810 -0.77019804 -0.09176391 0.0074322

T4 - T3 -0.6536689 -0.99288596 -0.31445183 0.0000411

*Identification of experimental apparatus or company sources is for information purposes only and does not imply endorsement by ARS, USDA.
